# Insights into Ni_3_TeO_6_ calcination *via in situ* synchrotron X-ray diffraction†

**DOI:** 10.1039/d4cp03765k

**Published:** 2024-11-01

**Authors:** Shubo Wang, Javier Fernández-Catalá, Qifeng Shu, Marko Huttula, Wei Cao, Harishchandra Singh

**Affiliations:** a Nano and Molecular Systems Research Unit, University of Oulu Oulu FIN-90014 Finland shubo.wang@oulu.fi harishchandra.singh@oulu.fi; b Materials Institute and Inorganic Chemistry Department, University of Alicante Ap. 99 E-03080 Alicante Spain; c Process Metallurgy Research Unit, University of Oulu FI-90014 Oulu Finland

## Abstract

The versatility of metal tellurate chemistry enables the creation of unique structures with tailored properties, opening avenues for advancements in a wide range of applications. However, precise nanoengineering of Ni_3_TeO_6_, a ceramic Ni tellurate with a broad variety of properties, like electrical, magnetic, photocatalytic and multiferroic properties, demands a deep understanding of the synthesis process, which is strongly influenced by experimental parameters. This study delves into the formation mechanism of Ni_3_TeO_6_ nanoparticles during calcination of hydrothermally produced precursors, using *in situ* synchrotron X-ray diffraction, complemented by post-mortem TEM and XPS, and thermal analysis. The results reveal a reaction sequence involving dehydration and dehydroxylation of stoichiometric Ni/Te oxyhydroxide coordinated by Te. This oxyhydroxide can be schematically represented by a formula of (3Ni/Te)(OOH)_4_·H_2_O. Subsequently, preferential nucleation of Ni_3_TeO_6_ occurs. Further calcination after full crystallization of Ni_3_TeO_6_ leads to the formation of a different Ni tellurate (NiTeO_4_) phase as an impurity. These findings clarify the reactions occurring during calcination of Ni/Te mixed precursors, which have frequently been inferred from empirical and post-mortem reports but not confirmed *via* comprehensive and *in situ* guided explorations.

## Abstract 2

This study reveals the sequence of reactions occuring during Ni_3_TeO_6_ calicination from hydrothermal precursor.

## Graphical abstract

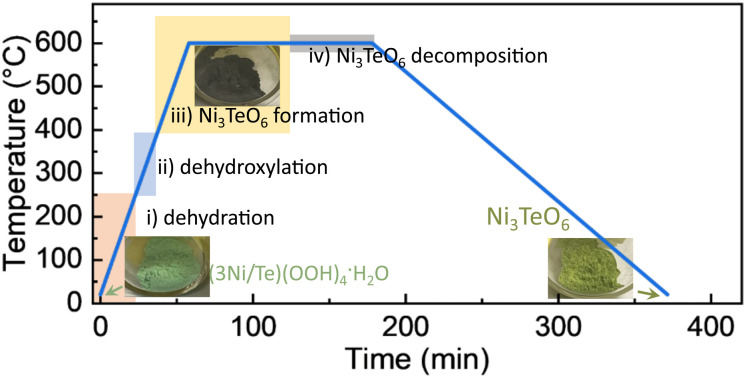

## 1. Introduction

There have been increasing interests in developing multifunctional nanomaterials due to the ability to tailor their properties through the precise control of composition, crystal structure, crystallinity, particle shape and size.^1–3^ To date, transition metal (M = Ni, Co, Mn, Cu) tellurates (MTOs) have emerged as highly attractive multifunctional nanomaterials. This is due to their diverse properties and broad applications, including electronics, energy, optics and catalysis.^4–7^ However, it has been reported frequently that a subtle variation in synthesis conditions could dramatically influence the phase purity, particle size, and crystalline structure of the resulting MTOs.^4–18^ This sensitivity and complexity in synthesis are likely due to the complex MTO chemistry. For example, six stable compounds of the Ni–Te–O system have already been reported so far, *i.e.* NiTeO_3_,^14^ NiTeO_4_,^15^ NiTe_2_O_5_,^16^ NiTe_6_O_13_,^17^ Ni_2_Te_3_O_8_^6,18^ and Ni_3_TeO_6_.^7–9,19^ Among these, Ni_3_TeO_6_ (denoted as NTO) is the longest known one, dating back to 1967.^19^ NTO can be synthesized using various methods, including solid state reactions,^7,8^ sol–gel processes^9^ and hydrothermal synthesis.^6^ These methods typically require mixing reagents with a Ni : Te ratio of 3 : 1 by solid state and wet chemistry (sol–gel or hydrothermal) methods, followed by a calcination step.^5–7,9–12^

Our previous studies demonstrated the possibility to tailor the morphology and functionality (antiferromagnetic, photoconductivity, and photocatalytic properties) of NTO using an efficient, cost-effective and versatile hydrothermal synthesis followed by calcination.^20–22^ This approach yielded NTO in the form of 2D nanosheets and nanoparticles (NP) by controlling the type and concentration of additives like CO(NH_2_)_2_ and NaOH during hydrothermal reactions. The overall reaction mechanism can be briefly described as follows:
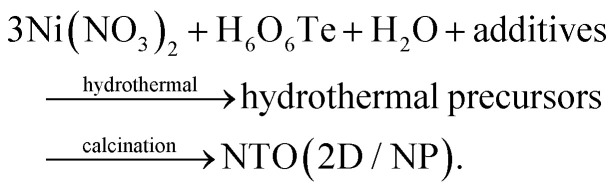


However, the details of intermediate formations remain unclear. For example, it is still unknown whether Ni_3_TeO_6_ nuclei form directly during hydrothermal synthesis or during the calcination stage, and what intermediates form between the reactant and the final product. Understanding these intermediate steps is critical for optimizing the synthesis process and achieving NTO products with enhanced functionalities.

To address this knowledge gap, we employed *in situ* synchrotron X-ray diffraction (SXRD) during calcination to track the phase transitions starting from the hydrothermally prepared NTO precursor. Additionally, post-mortem characterization techniques, such as transmission electron microscopy (TEM) and X-ray photoelectron spectroscopy (XPS), and thermal analysis were also used to corroborate the findings from SXRD. These combined data provide valuable insights into the synthesis and tailoring of nanostructured Ni_3_TeO_6_, potentially applicable to other Ni tellurates as well.

## 2. Materials and methods

**Fig. 1:**
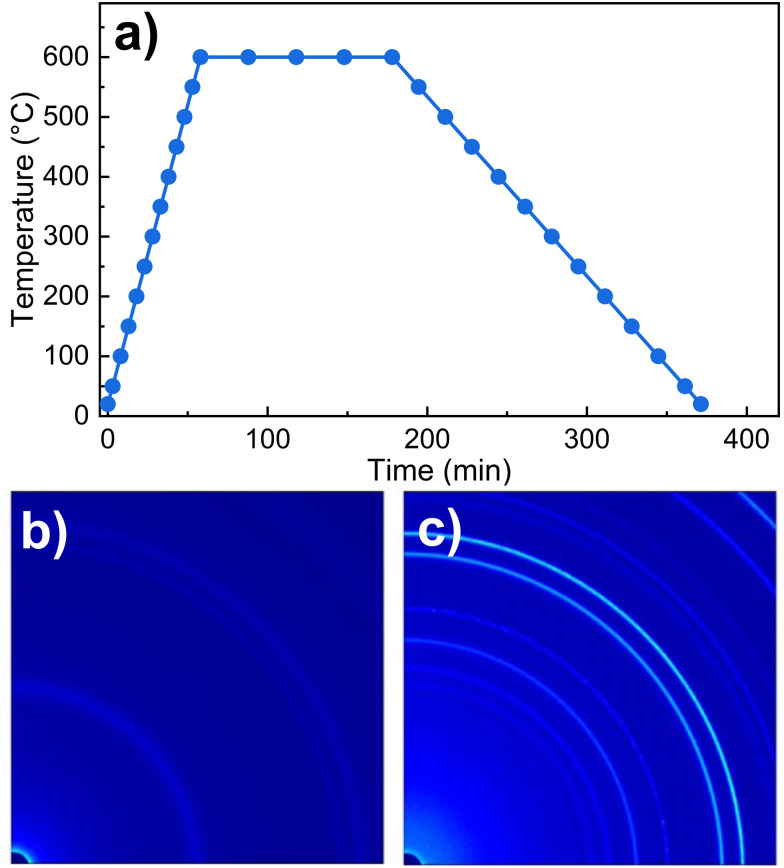
(a) A schematic temperature regime for *in situ* SXRD measurements, and the representative quarter of the collected 2D SXRD pattern for (b) NTO-hydro and (c) after calcination at 600 °C for 2 h, *i.e.* NTO-600. The circles in (a) highlight the temperatures where the SXRD patterns were collected.

The precursor prepared by hydrothermal synthesis (designated as NTO-hydro) was synthesized using reagents, such as Ni(NO_3_)_2_·6H_2_O, H_6_O_6_Te and NaOH, as described previously in ref. 22. Calcination of NTO-hydro was conducted using an *in situ* SXRD system at 70 keV (*λ* = 0.1779 Å) photons at the Brockhouse high energy wiggler beamline, Canadian light sources (CLS), Canada.^23^ The sample was heated from room temperature at a rate of 10 °C min^−1^ to a calcination temperature of 600 °C and held for 2 h (Fig. 1a). The calcination parameters were chosen based on previous successful syntheses of Ni_3_TeO_6_ nanomaterials.^20–22^ 2D diffraction data (Fig. 1b, c and Movie S1, ESI†) were collected using a transmission geometry,^24,25^ with an area detector positioned 1144 mm downstream of the sample. The calcinated powder was designated as NTO-600.

To complement the *in situ* SXRD analysis, the NTO-hydro precursor was additionally calcined in a muffle furnace at 450 °C for 5 min using the same heating rate as that used in the *in situ* experiment. The resulting powder was designated as NTO-450. Thermal analysis, using a STA449 F3 thermal analyzer (Netzsch-Gerätebau Gmbh) with Ar purge gas, was employed to determine the transition temperatures during calcination. The heating profiles were the same as that used in the SXRD measurements. TEM images for the NTO samples were acquired using a JEOL JEM-2200FS FETEM/STEM. XPS analyses were performed using a Thermo Fisher Scientific ESCALAB 250Xi XPS system with Avantage software for data acquisition and analysis. UV-vis spectra were collected using a Shimadzu UV-2600 spectrophotometer.

## 3. Results

**Fig. 2:**
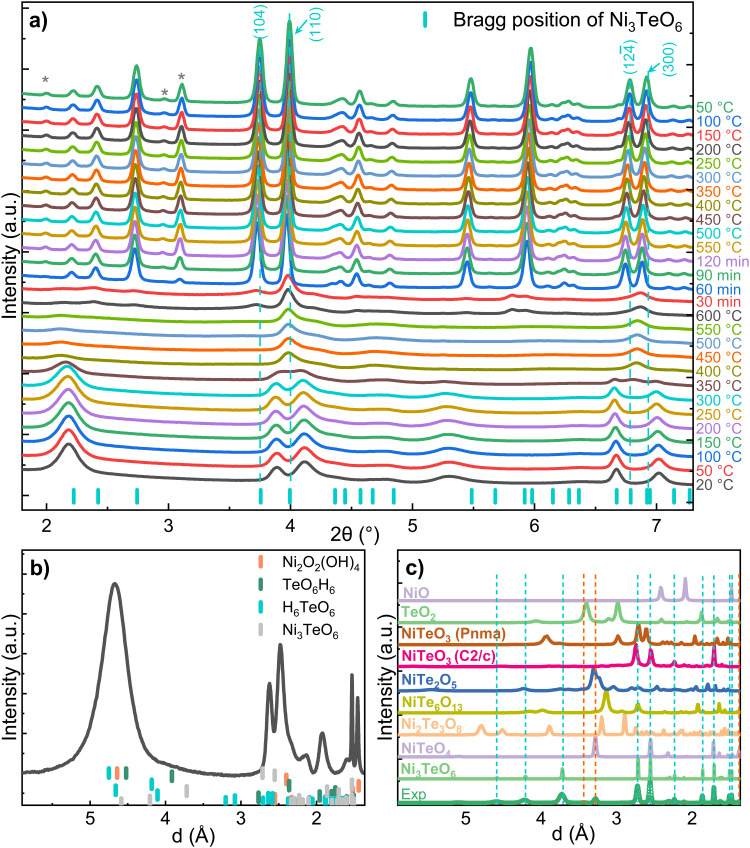
(a) SXRD profiles collected during the *in situ* heating and cooling cycle, indexing results of the diffractogram of (b) NTO-hydro, prior to calcination, and (c) NTO-600, after calcination. The 2*θ* angles in the *x*-axis in (a) were converted to *d* (interplanar spacing values) in (b) and (c). In (c), the solid lines depict the simulated powder XRD patterns of all the reported Ni–Te–O compounds mentioned above and possible decomposition products NiO (PDF 00-044-1159) and TeO_2_ (PDF 00-042-1365).

Fig. 2a displays the color-coded SXRD profiles collected within a 2*θ* range of 1.8–7.5°. The SXRD pattern of NTO-hydro exhibits relatively diffuse Debye–Scherrer rings (Fig. 1b), resulting in broad peaks in the corresponding 1D profiles. This indicates a short-range order or a relatively poor crystallinity in the material. Indexing of this pattern reveals that the primary constituent is nickel oxyhydroxide, Ni_2_O_2_(OH)_4_ (PDF 00-013-0229). No clear presence of crystalline Ni_3_TeO_6_ nuclei is observed due to overlapping Bragg peaks (Fig. 2b). However, the asymmetric peak shapes and the presence of shoulder peaks suggest the possible existence of minor phases.

During the ramping up stage, the broad peaks become less detectable. Concurrently, new peaks emerge at around 3.98° and 6.83°. By referring the indexing results in Fig. 2c, the peak at around 3.98° can be assigned to the (110) reflection of Ni_3_TeO_6_ with the *R*3 space group. This suggests the preferential nucleation of Ni_3_TeO_6_ crystals^21^ from the hydrothermal precursor. The latter peak at 6.83° is more asymmetric and splits into two sharp peaks at higher temperatures, corresponding to the (124̄) and (300) reflections of Ni_3_TeO_6_. Other diffraction peaks of Ni_3_TeO_6_ appear with increasing temperature and time.

After 60 min of isothermal calcination at 600 °C, well-crystalline Ni_3_TeO_6_ (*a* = *b* = 5.124 Å, *c* = 13.844 Å, *c*/*a* = 2.702, *α* = *β* = 90°, *γ* = 120°) is successfully obtained. The (110) reflection at 3.98° remains the strongest peak, contrasting with the database reference (PDF 04-009-2820, Fig. 2c) where the strongest peak is the (104) reflection at 3.72°. This comparative result indicates that the synthesized Ni_3_TeO_6_ shows (110) as the preferred orientation in this study. Meantime, a weak peak (marked by *) becomes detectable, indicating the presence of an impurity phase. The intensity of this peak increases with longer calcination duration up to 120 min. No significant changes are observed during the cooling stage. The impurity phase, exhibiting peaks at 2.01, 2.91 and 3.12° (corresponding to *d* values of 0.509, 0.344 and 0.327 nm), has been identified as a different nickel tellurate NiTeO_4_ with a monoclinic *P*121/*c*1 space group (*a* = 6.114 Å, *b* = 4.667 Å, *c* = 5.575 Å, *α* = *γ* = 90°, and *β* = 123.44°) as shown in Fig. 2c, with better visibility in Fig. S1a (ESI†). Quantitative analysis using the Rietveld refinement (Fig. S1b, ESI†) shows that the NiTeO_4_ phase content increases with increasing calcination durations and finally reaches ∼10.4 wt% (Table S1, ESI†). It again confirms a good fit to *R*3 Ni_3_TeO_6_ with lattice constants of *a* = *b* = 5.099 Å and *c* = 13.754 Å (*c*/*a* = 2.697) and *P*121/*c*1 NiTeO_4_ with lattice constants of *a* = 6.088 Å, *b* = 4.638 Å and *c* = 5.545 Å at room temperature, corresponding to thermal expansion coefficients of 5.29 × 10^−5^ °C^−1^ along the *a* and *b* axes and 1.51 × 10^−4^ °C^−1^ along the *c* axis for Ni_3_TeO_6_ and 4.82 × 10^−5^, 5.01 × 10^−5^, and 5.24 × 10^−5^ °C^−1^ along the *a*, *b*, and *c* axes for NiTeO_4_ during the cooling stage, respectively (Table S1, ESI†).

**Fig. 3:**
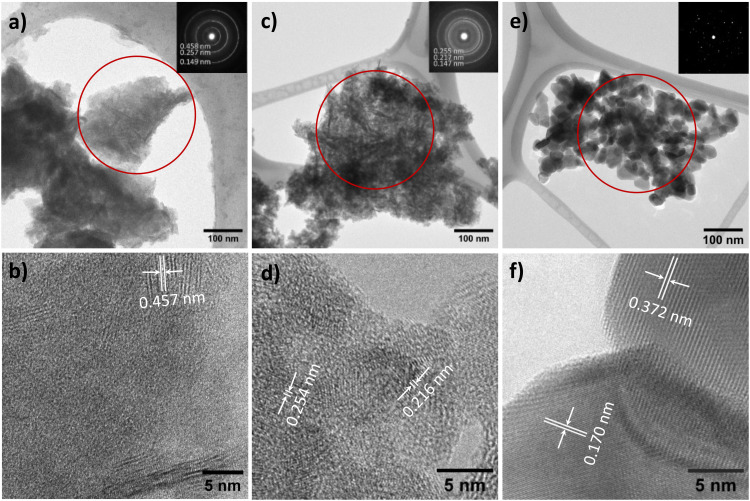
Bright field TEM (upper panel) and HRTEM (lower panel) images for (a) and (b) NTO-hydro, (c) and (d) NTO-450, and (e) and (f) NTO-600 samples. Insets show the SAED patterns (contrast was enhanced for clarity) from the circled areas.

**Fig. 4:**
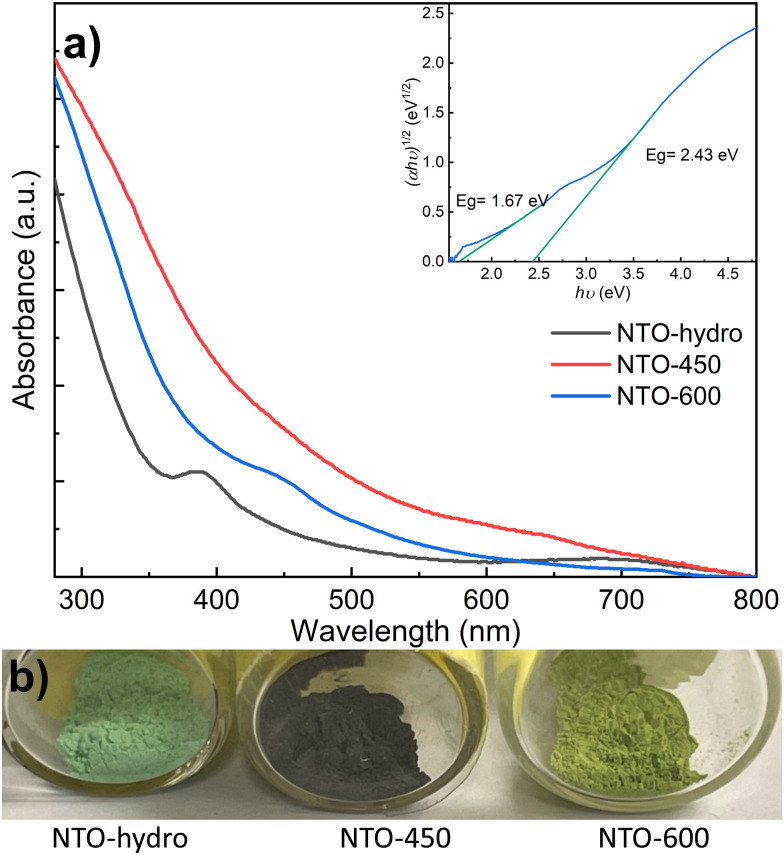
(a) UV-vis absorbance spectra and (b) digital images of NTO-hydro, NTO-450 and NTO-600. The inset of (a) depicts the Tauc plot for NTO-600.

The TEM image reveals the characteristic morphology of NTO-hydro, *i.e.* flake-like shapes composed of numerous agglomerated nanoparticles and nanosheets (Fig. 3a). This NTO-hydro exhibits a characteristic greenish color and weak visible light absorption (Fig. 4), which is consistent typically with Ni (oxy)hydroxides.^26^ The corresponding selected area electron diffraction (SAED) in Fig. 3b displays relatively diffuse rings centered at *d*-spacings of 0.458, 0.257 and 0.149 nm, along with a few discrete diffraction spots scattered along the rings. These features are in good agreement with (001), (110) and (020) interplanar spacings of Ni_2_O_2_(OH)_4_ (Fig. 2b). No Te containing phase can be definitively resolved by TEM. This might be attributed to (i) the poor crystallinity of the sample and (ii) the use of a relatively low beam density to minimize beam damage to hydroxides.^27^ However, the uniform distribution of Ni, Te and O elements observed in the STEM elemental mapping (Fig. S2, ESI†) indicates the presence of Te within the sample. Based on this evidence and previous report on Te incorporation with edge metal atoms in transition metal hydroxides,^28^ it is reasonable to conclude that Te might incorporate into Ni_2_O_2_(OH)_4_, forming Te–OH and/or Te–O bonds.

In contrast to NTO-hydro, the morphology of NTO-450 (obtained after calcination at 450 °C with 5 min holding) barely changes (Fig. 3c). However, its color is seen to change significantly to dark olive or grey, exhibiting the strongest visible light absorption (Fig. 4). The SAED pattern (inset of Fig. 3c) reveals diffraction rings at *d*-spacings of 0.255, 0.217 and 0.147 nm, corresponding to the (110), (021) and (300) of Ni_3_TeO_6_. This represents the disappearance of Te incorporated Ni_2_O_2_(OH)_4_ and formation of Ni_3_TeO_6_. The HRTEM image in Fig. 3d reveals a much longer-range order compared to NTO-hydro, indicating the improved crystallinity of the material. It further suggests that the crystalline Ni_3_TeO_6_ nanoparticles are likely only a few nanometers in size. The color of this NTO-450 thus can be ascribed to the formation of this ultrafine nanocrystalline oxide structure.^29,30^

After calcination, NTO-600 exhibits heterogeneous nanoparticle morphology, as shown in Fig. 3e. It results in discrete diffraction spots in the corresponding SAED pattern. The HRTEM image (Fig. 3f) confirms the high crystallinity and presence of the Ni_3_TeO_6_ crystalline phase. The strong visible light absorption of NTO-600, starting at around 520 nm (Fig. 4a), is attributed to its band gap (*E*_g_ ≈ 2.43 eV) characteristic of Ni_3_TeO_6_. This relatively small *E*_g_ is induced by the excitation of photoelectrons from O 2p to Ni 3d orbitals, consistent with previous reports.^9,22^ The second band at 1.67 eV, corresponding to the broad absorption beyond 520 nm, aligns with electron transition from the occupied Ni 3d to empty Ni 3d orbitals.^31^

SXRD collects the bulk information as the high energy X-rays penetrate the entire sample volume. In contrast, XPS is surface-sensitive. The sampling depth of XPS is limited to approximately 3*λ* (*λ* is the inelastic mean free path for photoelectrons),^32^ estimated to be within 2.8–3.5 nm of the outmost surfaces for the studied materials with the applied Al Kα X-ray source. Considering the sample morphologies in Fig. 3, the XPS signal likely contains the relatively bulk information from NTO-hydro and NTO-450, while the signal from NTO-600 reflects the surface characteristics of the crystalline nanoparticles. Therefore, directly comparing the compositional information obtained from peak deconvolution of XPS spectra would be misleading. Consequently, only the fitted peak characteristics are listed in Table S2 (ESI†).

**Fig. 5:**
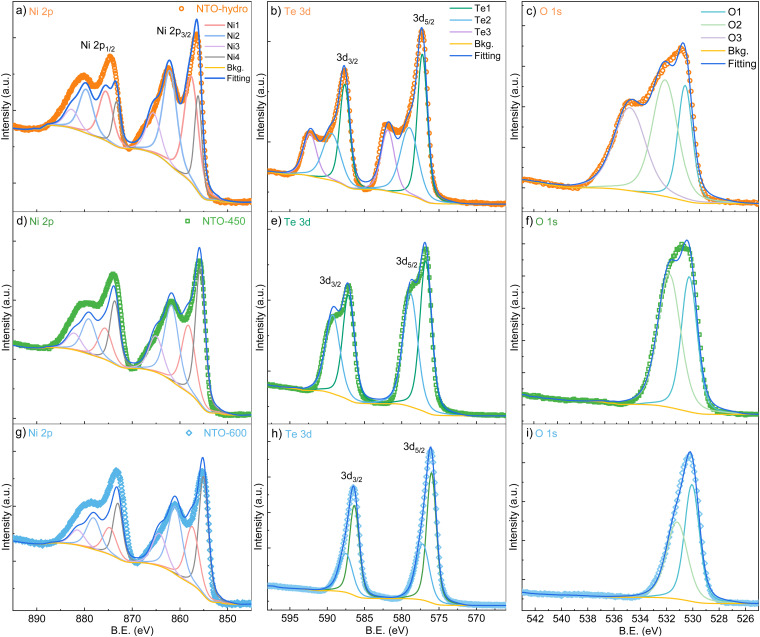
The peak deconvolution for high-resolution XPS spectra of (a), (d) and (g) Ni 2p, (b), (e) and (h) Te 3d and (c) (f) and (i) O 1s core levels for (a)–(c) NTO-hydro, (d)–(f) NTO-450 and (g)–(i) NTO-600, respectively.

The XPS survey spectra of all three samples show all the expected peaks without any additional or impurity elements (Fig. S3, ESI†). High-resolution spectra in Fig. 5 reveal that the asymmetric peaks for Te and O narrow down significantly after calcination, while peaks for Ni exhibit no significant changes. This observation prompts us to focus on the Te 3d (Fig. 5b) and O 1s (Fig. 5c) spectra first. To achieve optimum fitting while avoiding overfitting, a minimum number of components were also employed for these spectra. Notably, the Te 3d and O 1s of NTO-hydro required one additional doublet/singlet peak, compared to the calcinated NTO-450 and NTO-600 samples.

The Te 3d core level spectra exhibit two 3d_5/2_ and 3d_3/2_ spin orbit doublets with a characteristic peak area ratio of 3 : 2, despite their broadness. For NTO-hydro, the broadest Te 3d level is optimally fitted with three components at 577.1, 578.8 and 581.7 eV for the 3d_5/2_ level, labelled as Te1, Te2 and Te3, respectively. In contrast, NTO-450 and NTO-600 require only two components, Te1 and Te2, at 576.6 eV and 578.6 eV (NTO-450) and 576.1 eV and 577.3 eV (NTO-600) for the 3d_5/2_ level, respectively. These binding energies can be compared to reference compounds, *i.e.* TeO_2_ (Te^4+^) at 576.1 eV, Te(OH)_6_ (Te^6+^) at 576.6 eV, and TeO_3_ (Te^6+^) at 577.3 eV.^33^ The difference in binding energy, nearly 1.0 eV, between the fitted Te components in NTO-hydro and NTO-600 (Table S2, ESI†) suggests that components with the same name likely represent different Te species in these samples. For the crystalline NTO-600, Te1 and Te2 suggest the presence of a dominant Te^6+^ and a minor Te^4+^ species, consistent with our previous study.^22^ In NTO-hydro and NTO-450, Te1 corresponds to a Te^6+^ species, while Te2 and Te3 suggest a more complex bonding environment for Te involving oxygen and hydroxyl groups. Qualitatively, the broader peaks and higher binding energies in NTO-hydro and NTO-450 indicate a higher oxidation state of Te (≥6+) with few electrons per atom. Calcination promotes electron enrichment around Te, leading to a lower oxidation state (between 6+ and 4 +) and a shift to lower binding energies.

The changes of the O 1s spectra align with those of the Te 3d spectra (Fig. 5c). The O 1s in NTO-hydro must be fitted with three, O1, O2 and O3, components centered at 531.0, 532.7, and 535.7 eV, respectively. The first two components correspond to oxide lattice oxygen, hydroxyl groups and/or oxygen vacancies,^34^ which remain after calcination. The third component can be attributed to a mixture of adsorbed molecular water and free or vapor phase water,^35^ both of which disappear along with the aforementioned Te3 component during calcination. Upon calcination, the O1 and O2 peaks become narrower and shift towards lower binding energies. Notably, the binding energy difference for O1 (representing oxide lattice oxygen) between NTO-hydro and NTO-600 is only 0.2 eV (Table S2, ESI†), indicating a relatively consistent chemical environment due to the presence of short-range or long-range ordered oxides. A more significant change is seen in O2, which represents the hydroxyl groups and/or oxygen vacancies. This suggests that the enhanced crystallinity and atomic order in the material are associated with the dehydroxylation reaction.

The Ni 2p spectra demonstrate complex line shapes with satellite features at higher binding energy just beside the main peaks (Fig. 5a). Poorer fitting can be found at the binding energy region corresponding to the Ni 2p_1/2_ level compared to Ni 2p_3/2_, due to the overlap with Te 3p_1/2_.^36^ However, the line shapes of this core level are relatively consistent across the three samples. Smaller binding energy variations among the four fitted components are seen compared to Te 3d and O 1s levels, although intensity variations exist (Fig. 5 and Table S2, ESI†). These four nickel components (Ni1–4) can be assigned to Ni^2+^ species in Ni–O (Ni1) and Ni–OH (Ni2) bonding configurations and their respective satellites (Ni3 and 4),^37–40^ based on their binding energies, peaks, FWHM and the O 1s deconvolution. This agrees well with Ni in a 2+ oxidation state. However, the shift towards lower binding energy upon calcination suggests an interaction between Ni and Te, particularly with the Te3 and O3 components identified earlier. This interaction is likely responsible for the presence of Ni with a higher oxidation state (4+) within Ni_2_O_2_(OH)_4_ in NTO-hydro.

**Fig. 6:**
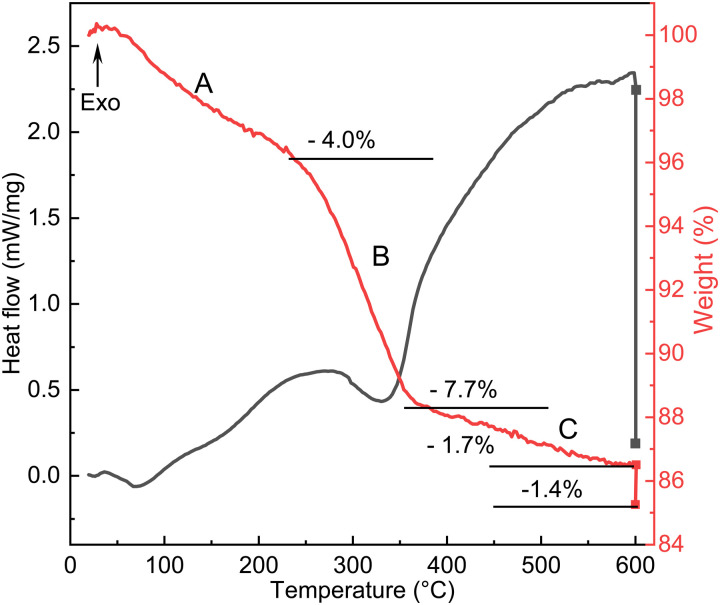
Heat flow and mass change curves during the calcination of NTO-hydro.

To understand the reactions occurring during the calcination step, we have also measured the heat flow and mass changes, as plotted in Fig. 6. The results are consistent with the SXRD data (Fig. 2), particularly the characteristic peak of the oxyhydroxide phase located around 2.2° (Fig. 2b), and its peak characteristics are summarized in Table S3 (ESI†). This reveals that calcination of NTO-hydro to NTO-600 is a complex, multistage process. The first stage (A) involves a weight loss of 4.0 wt% below 250 °C, likely attributed to the evaporation of absorbed and intercalated water (the O3 component in XPS spectra) coordinated mainly with Te (the Te3 component) in NTO-hydro. This dehydration process results in the broad, shallow endothermic grooves in the heat flow curve. Correspondingly, the oxyhydroxide diffraction exhibits a slight left shift in the peak center due to lattice expansion. There are only minor changes in the integrated peak area, but the peak intensity decreases while the peak width increases (Table S3, ESI†). Stage B follows, characterized by an endothermic process that causes a slight dip in the heat flow at 280–360 °C, coinciding with a weight loss of 7.7 wt%. The peak located initially at around 2.18° becomes significantly weaker and narrower, with a pronounced left-shift peak center. In the temperature range of 360 °C to 600 °C (stage C), the weight loss curve becomes smoother (1.7 wt%), while the heat flow curve displays a continuous exothermic characteristic. Meantime, the characteristic oxyhydroxide peak becomes progressively weaker and narrower, while its peak centre shifts left first and then right (Table S3, ESI†). These changes indicate the nucleation and growth of Ni_3_TeO_6_ (Fig. 1), which results in a sharp defined peak at 2.204°, corresponding to the (003) reflection of Ni_3_TeO_6_ after 60 min isothermal calcination at 600 °C (Fig. 2a). Further isothermal calcination up to 2 h leads to a further 1.4 wt% mass loss, which could possibly suggest the decomposition of Ni_3_TeO_6_.^8,13^

## 4. Mechanism of nano-Ni_3_TeO_6_ calcination

While the SXRD profiles in Fig. 2 show no phase transition before 400 °C, some reactions likely occur according to the thermal analysis. It has been reported that the nickel hydroxide decomposition to oxide occurs at around 320 °C.^41,42^ Therefore, stage B can be attributed to the loss of hydroxyls through

1 Ni_2_O_2_(OH)_4_ → Ni_2_O_4_ + 2H_2_O,

and less possibly stepwise decomposition of Te(OH)_6_ at 130–215 °C and onwards until about 400 °C:^43^

2 Te(OH)_6_ → H_2_TeO_4_ + 2H_2_O → TeO_3_ + 3H_2_O

The theoretical weight loss values calculated from Eqs. 1 and 2, 16.5 and 23.5 wt%, are significantly higher than measured values. Such deviations, again, confirm the incorporation of Te into the Ni oxyhydroxide structure. This results in a more complicated layered structure of NTO-hydro with a short-range order, resembling orthorhombic Ni_2_O_2_(OH)_4_ rather than Ni(OH)_2_.^40^

Assuming a stoichiometric conversion of Te incorporated Ni oxyhydroxides to 3NiO/TeO_3_ for the subsequent formation Ni_3_TeO_6_, eqn (1) can be modified as follows:

3 3(Ni_2_O_2_(OH)_4_) + 2 incorporated Te → 2(3NiO/TeO_3_) + 6H_2_O.

Here, the calculated weight loss, 11.9 wt%, is still higher than the experimental value (7.7 wt%) for stage B. However, it is close to a summed weight loss of 11.7 wt% for stages A and B. This suggests that the principal oxyhydroxide component in NTO-hydro can be represented as a formula of (3Ni/Te)O_4_(OH)_4_·H_2_O, or (3Ni/Te)(OOH)_4_·H_2_O, coordinated by Te as evidenced by XPS data (Fig. 5).

Based on these observations, we propose the following reaction sequence for NTO-hydro during calcination:

4 (3Ni/Te)(OOH)_4_·H_2_O (orthorhombic) → (3Ni/Te)(OOH)_4_ + H_2_O

It occurs in the temperature range of room temperature to about 260 °C, resulting in a weight loss of 4.0 wt% (stage A). Following ramps to 360 °C results in:

5 (3Ni/Te)(OOH)_4_ (orthorhombic) → (3Ni/Te)O_6_+ 2H_2_O

This dehydroxylation reaction results in a weight loss of 7.9% (stage B). It leads to a right-shift and broadening of the diffraction peaks in the 2*θ* range above 2.5° and left-shift and narrowing of the characteristic oxyhydroxide peak, without the emergence of a new phase (Fig. 2). This suggests that the resulting oxide maintains the same crystal structure as oxyhydroxide.

In the temperature range of 360 °C to 600 °C (stage C), preferred nucleation of Ni_3_TeO_6_ occurs:

6 (3Ni/Te)O_6_ (orthorhombic)→ Ni_3_TeO_6_

Complete transition to well-crystalline Ni_3_TeO_6_ is achieved after 30 to 60 min isothermal calcination at 600 °C. Extended calcination leads to the formation of impurities, which is likely due to the relatively low oxygen partial pressure in the thin quartz capillary compared to a more spacious muffle furnace with air circulation.^22^ This highlights the importance of an oxygen rich environment, appropriate elevated temperatures, and a controlled calcination duration to avoid impurities during the synthesis of pure Ni_3_TeO_6_ nanomaterials.

## 5. Conclusions

In conclusion, this study investigated the formation of Ni_3_TeO_6_ nanoparticles from a Ni/Te mixture precursor prepared by a hydrothermal method. *In situ* SXRD, complemented by TEM, XPS and thermal analysis, provided insights into the calcination process. The results reveal that the hydrothermally obtained powder precursor exhibits a short-range order and a nickel oxyhydroxide structure with incorporation of Te, schematically represented as (3Ni/Te)(OOH)_4_·H_2_O. Calcination of this complex oxyhydroxide is a multistage process, starting with dehydration followed by dehydroxylation and nucleation of Ni_3_TeO_6_. A Ni : Te stoichiometric ratio of 3 : 1 remains consistent with the initial aqueous solution, and preferential nucleation from the (110) plane of Ni_3_TeO_6_ is observed. However, an impurity tellurate phase appears after complete crystallization of Ni_3_TeO_6_ during the *in situ* SXRD experiment. This is likely due to the deviations from the ideal calcination environment. We believe that this study sheds light on the understanding of nanostructured Ni_3_TeO_6_ from its precursors during calcination. This knowledge can be applied to optimize the process and manipulate the size and morphology of Ni_3_TeO_6_ and other metal tellurates.

## Data availability

Microscopic data supporting the finding are available within the article, and the raw SXRD profiles at different temperatures, and also the raw XPS, UV-Vis and thermal analysis spectra, are available within its ESI.†

## Conflicts of interest

The authors declare that they have no known competing financial interests or personal relationships that could have appeared to influence the work reported in this paper.

## Supplementary Material

CP-026-D4CP03765K-s001

CP-026-D4CP03765K-s002
