# Supplementary material for: Insights into Ni3TeO6 calcination via in situ synchrotron X-ray diffraction
Source: Phys Chem Chem Phys. 2024 Nov 1;26(46):28913–21. doi: 10.1039/d4cp03765k (PMC11563211; doi:10.1039/d4cp03765k)
Supplement: CP-026-D4CP03765K-s001 [file CP-026-D4CP03765K-s001.pdf]

Supplementary Information (SI) for Physical Chemistry Chemical Physics.
This journal is © the Owner Societies 2024

Supplementary Material

Insights into Ni_3TeO_6 calcination via in situ synchrotron X-ray diffraction

Shubo Wang [a,], Javier Fernández-Catalá [a,b], Qifeng Shu [c], Marko Huttula [a], Wei Cao [a], Harishchandra Singh [a,*]*

[a] Nano and Molecular Systems Research Unit, University of Oulu, FIN-90014, Oulu, Finland

Materials Institute and Inorganic Chemistry Department, University of Alicante, Ap. 99, E-03080 Alicante, Spain.

**Corresponding authors. E-mails: shubo.wang@oulu.fi. harishchandra.singh@oulu.fi.*

Detailed experimental methods

In situ SXRD measurements were conducted at the Brockhouse high energy wiggler beamline, Canadian Light Source (CLS), Canada. The powder NTO-hydro sample, loaded in a Φ0.9 mm (inner dimension) quartz capillary, was heated using a flow-cell furnace [1] at ambient atmosphere but without air circulation, simulating the calcination procedure in our lab using muffle furnace. A 2D Perkin Elmer area detector (200 × 200 μm^2 pixel size, 40 × 40 cm^2 in area) placed behind the sample allowed 2D XRD diffraction pattern in transmission mode. Wavelength of the monochromatic X-ray beam (100 µm vertical and 200 µm horizontal) and detector to sample distance were 0.1779 Å and 1144 mm, calibrated using a standard Ni powder calibrants with known lattice parameter. Diffraction patterns were collected a 50 °C step manner with an exposure time of 2.0 s that was much shorter than the continuous temperature ramping rate. Therefore, each frame corresponded to the exact specific temperature simultaneously recorded by the thermocouple. The obtained 2D diffraction patterns were integrated into 1D profiles using open-source GSAS-II software [2].

For DSC measurement, temperature and accuracy calibration were carried out using high-purity metals with well-known melting pointing as reference norms. During the measurement, Pt

crucibles with a Pt lid were employed to minimize the loss of materials, and alumina powders was employed as the inert reference material. NTO-hydro material was weighed to be 19.7 mg prior to measurement. The measured DSC heat flow and mass loss is representative of the energy (in unit of mW), and mass change (wt.% relative to the starting weight) as a function of temperature.

XPS measurements were performed with Al Kα using Thermo Fisher Scientific ESCALAB 250Xi XPS System. Energy calibration of the XPS was performed by using the C *1s* peak at 284.8 eV.

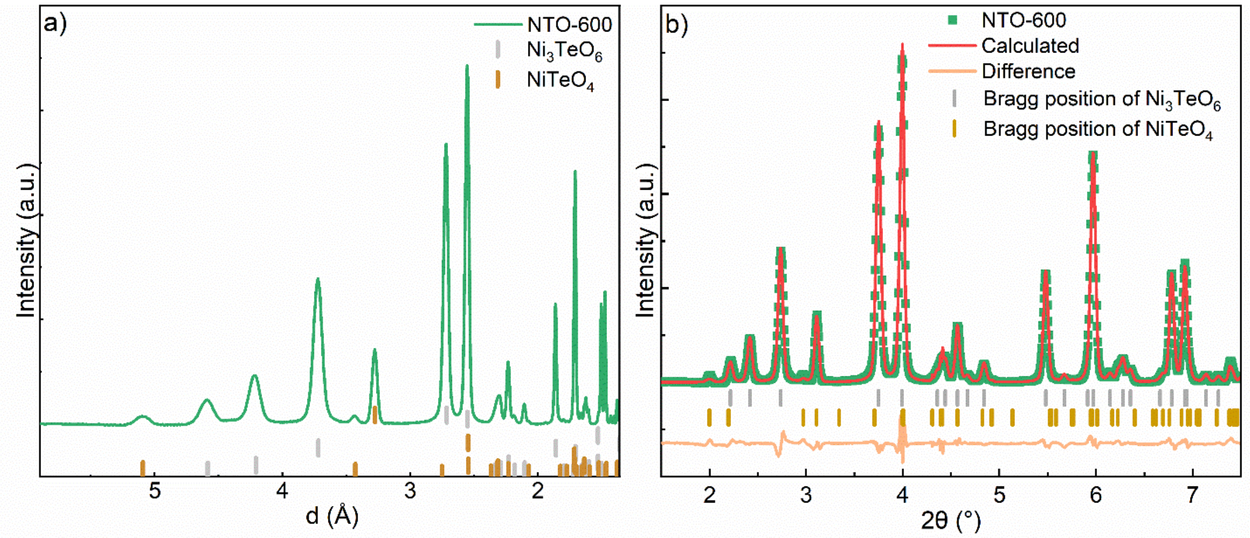

Fig. S1 (a) Enlarged view of the indexing results for the SXRD profile of NTO-600, and (b) corresponding Rietveld refinement.

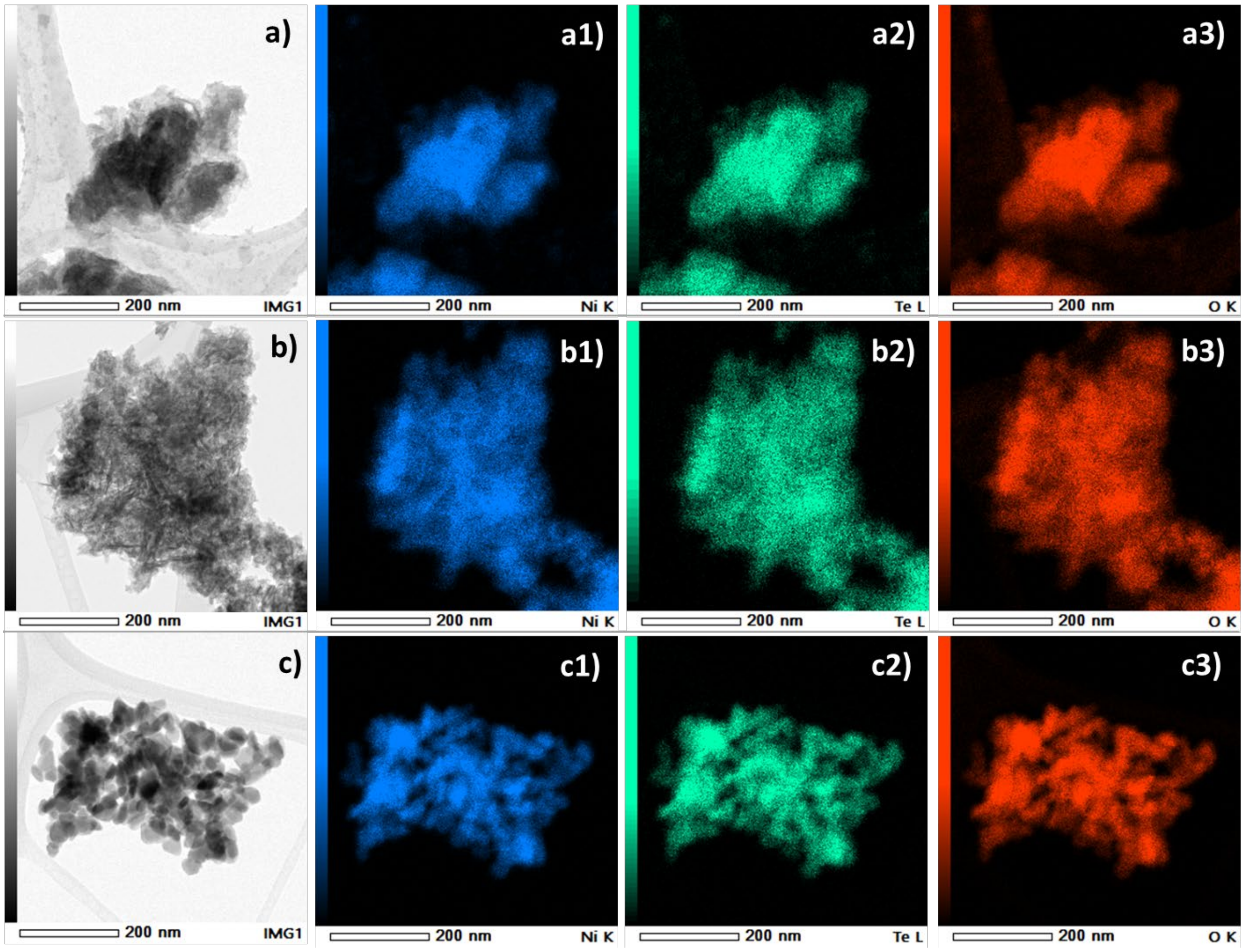

Fig. S2 STEM image and corresponding Ni (green), Te (blue), and O (red) EDS mapping results for (a) NTO_hydro, (b) NTO_450, and (c) NTO_600.

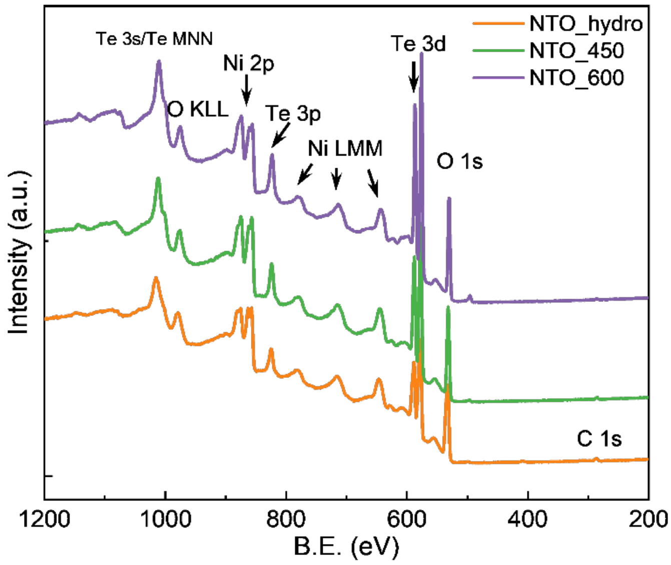

Fig. S3 XPS survey spectra for all the three samples.

Table S1. Quantified phase fractions and lattice constants of Ni_3TeO_6 and $NiTeO_4$ impurity phase at different temperatures.

<table>
<tr><td rowspan="3" colspan="2">Temperature (°C) / Time</td><td colspan="3">Ni_3TeO_6</td><td colspan="4">$NiTeO_4$</td></tr>
<tr><td rowspan="2">Fraction (wt%)</td><td colspan="2">Lattice constants (Å)</td><td rowspan="2">Fraction (wt%)</td><td colspan="3">Lattice constants (Å)</td></tr>
<tr><td>a=b</td><td>c</td><td>a</td><td>b</td><td>c</td></tr>
<tr><td rowspan="5">Isothermal holding at 600 °C</td><td>0 min</td><td>100.0</td><td>-</td><td>-</td><td>0</td><td>-</td><td>-</td><td>-</td></tr>
<tr><td>30 min</td><td>100.0</td><td>-</td><td>-</td><td>0</td><td>-</td><td>-</td><td>-</td></tr>
<tr><td>60 min</td><td>94.8</td><td>5.124</td><td>13.845</td><td>5.2</td><td>-</td><td>-</td><td>-</td></tr>
<tr><td>90 min</td><td>92.1</td><td>5.124</td><td>13.844</td><td>7.9</td><td>6.114</td><td>4.667</td><td>5.575</td></tr>
<tr><td>120 min</td><td>89.6</td><td>5.128</td><td>13.841</td><td>10.4</td><td>6.116</td><td>4.667</td><td>5.574</td></tr>
<tr><td rowspan="11">Air cooling to room temperature</td><td>550</td><td>89.6</td><td>5.126</td><td>13.833</td><td>10.4</td><td>6.113</td><td>4.664</td><td>5.572</td></tr>
<tr><td>500</td><td>89.6</td><td>5.123</td><td>13.825</td><td>10.4</td><td>6.111</td><td>4.661</td><td>5.569</td></tr>
<tr><td>450</td><td>89.6</td><td>5.120</td><td>13.818</td><td>10.4</td><td>6.108</td><td>4.659</td><td>5.566</td></tr>
<tr><td>400</td><td>89.6</td><td>5.117</td><td>13.809</td><td>10.4</td><td>6.106</td><td>4.656</td><td>5.563</td></tr>
<tr><td>350</td><td>89.6</td><td>5.114</td><td>13.801</td><td>10.4</td><td>6.104</td><td>4.653</td><td>5.559</td></tr>
<tr><td>300</td><td>89.6</td><td>5.112</td><td>13.794</td><td>10.4</td><td>6.101</td><td>4.651</td><td>5.558</td></tr>
<tr><td>250</td><td>89.6</td><td>5.109</td><td>13.786</td><td>10.4</td><td>6.098</td><td>4.649</td><td>5.555</td></tr>
<tr><td>200</td><td>89.6</td><td>5.107</td><td>13.779</td><td>10.4</td><td>6.096</td><td>4.646</td><td>5.553</td></tr>
<tr><td>150</td><td>89.6</td><td>5.105</td><td>13.773</td><td>10.4</td><td>6.094</td><td>4.643</td><td>5.549</td></tr>
<tr><td>100</td><td>89.6</td><td>5.102</td><td>13.766</td><td>10.4</td><td>6.092</td><td>4.642</td><td>5.548</td></tr>
<tr><td>RT</td><td>89.6</td><td>5.099</td><td>13.754</td><td>10.4</td><td>6.087</td><td>4.638</td><td>5.545</td></tr>
<tr><td colspan="3">Coefficient of thermal expansion (10^{-5} °C^{-1})</td><td>5.29</td><td>15.1</td><td>-</td><td>4.82</td><td>5.01</td><td>5.24</td></tr>
</table>

Table S2. The deconvolution of Ni *2p,* Te *3d* and O *1s* XPS spectra: the binding energy (B.E., eV), FWHM (full width at half maximum, eV) of peaks.

Chemical state	Core levels		NTO-hydro	NTO-450	NTO-600
Ni1	B.E.	$2p_{3/2}$	856.0	855.5	855.2
		$2p_{1/2}$	873.1	873.5	873.1
	FWHM (eV)		1.8	2.4	2.4
Ni2	B.E.	$2p_{3/2}$	858.4	858.0	857.5
		$2p_{1/2}$	875.7	875.7	874.7
	FWHM (eV)		3.5	3.3	3.3
Ni3	B.E.	$2p_{3/2}$	861.9	861.5	861.1
		$2p_{1/2}$	879.5	878.9	878.2
	FWHM		3.5	3.5	3.5
Ni4	B.E.	$2p_{3/2}$	865.4	864.8	864.4
		$2p_{1/2}$	882.5	881.9	881.5
	FWHM		3.5	3.5	3.5
Te1	B.E.	$3d_{5/2}$	577.1	576.6	576.1

		$3d_{3/2}$	587.5	586.9	586.5
	FWHM		2.0	1.9	1.5
Te2	B.E.	$3d_{5/2}$	578.8	578.6	577.3
		$3d_{3/2}$	589.2	589.0	587.6
	FWHM		2.8	2.3	2.1
Te3	B.E.	$3d_{5/2}$	581.7		
		$3d_{3/2}$	592.3		
	FWHM		2.12		
O1	B.E.	1s	531.0	530.9	530.8
	FWHM		1.66	1.59	1.35
O2	B.E.	*1s*	532.7	532.3	531.3
	FWHM		2.52	2.27	1.9
O3	B.E.	*1s*	535.7		
	FWHM		3.15		

Table S3. Peak characteristics of the diffraction peak located around 2.2° (2θ range of 1.9-2.4°) for the SXRD patterns.

Temperature	Associated reaction	Intensity	Integrated area	Peak center	FWHM
RT	Stage A: dehydration	2228	443	2.179	0.175
50 °C		2223	436	2.180	0.175
100 °C		2219	441	2.177	0.176
150 °C		2161	441	2.174	0.181
200 °C		2099	434	2.172	0.186
250 °C		2006	417	2.169	0.189
300 °C	Stage B: dehydroxylation	1705	346	2.166	0.186
350 °C		833	158	2.150	0.179
400 °C		305	50	2.132	0.164
450 °C	Stage C: Ni_3TeO_6 formation	186	30	2.119	0.157
500 °C		151	23	2.156	0.156
550 °C		119	17	2.112	0.139
600 °C		69	7.6	2.125	0.120
600 °C / 30 min		50	5.7	2.132	0.123
600 °C / 60 min		428	26	2.204	0.056

NTO_calcination SXRD patterns.mp4

Movie. S1 Evolution of 2D diffraction patterns as a function of temperature.

Reference

[1] P.J. Chupas, K.W. Chapman, C. Kurtz, J.C. Hanson, P.L. Lee, C.P. Grey, A versatile sample-environment cell for non-ambient X-ray scattering experiments, J. Appl. Crystallogr. 41 (2008) 822–824. https://doi.org/10.1107/S0021889808020165.

[2] B.H. Toby, R.B. Von Dreele, GSAS-II : the genesis of a modern open-source all purpose crystallography software package, J. Appl. Crystallogr. 46 (2013) 544–549. https://doi.org/10.1107/S0021889813003531.
