# Supplementary material for: Insights into Ni3TeO6 calcination via in situ synchrotron X-ray diffraction
Source: Phys Chem Chem Phys. 2024 Nov 1;26(46):28913–21. doi: 10.1039/d4cp03765k (PMC11563211; doi:10.1039/d4cp03765k)
Supplement: CP-026-D4CP03765K-s002 [file CP-026-D4CP03765K-s002.zip › Movie_S1_Evolution_of_2D_diffraction_patterns_as_a_function_of_temperature.docx]

**Movie. S1** Evolution of 2D diffraction patterns as a function of temperature.
